# Gender-differences in age-related changes of corneal astigmatism in Korean cataract patients

**DOI:** 10.1186/s12886-018-1001-1

**Published:** 2019-01-24

**Authors:** Hyojin Kim, Youngju An, Choun-Ki Joo

**Affiliations:** 1 Department of Visual Optics, Division of Health Science and Graduate School of Health and Welfare, Baekseok University, Cheonan, South Korea; 2 Catholic Institute for Visual Science, College of Medicine, The Catholic University of Korea, Seoul, South Korea; 3 Department of Ophthalmology and Visual Science, Seoul St. Mary’s Hospital, College of Medicine, The Catholic University of Korea, #505 Banpo-Dong, Seocho-Gu, Seoul, 137-040 South Korea

**Keywords:** Against-the-rule, Aging, Corneal astigmatism, Sex, With-the-rule

## Abstract

**Background:**

This cross-sectional study investigated age-related changes in anterior, posterior, and total corneal astigmatism and evaluated sex differences in corneal astigmatism with increasing age in cataract patients.

**Methods:**

This study evaluated eyes with cataracts from May 2009 and July 2013. All eyes underwent a complete ophthalmological examination and corneal Scheimpflug imaging by a Pentacam camera (Oculus, Wetzlar, Germany). Anterior, posterior, and total corneal astigmatism were determined. Power vector J_0_ and linear regression analyses were determined and compared with respect to age and sex.

**Results:**

Four hundred and fifteen patients (217 men, 198 women) aged 20–89 years were evaluated. For anterior corneal astigmatism, 100% of patients who were 20–39 years old had with-the-rule (WTR) in both sexes. WTR was significantly lower in patients 80–89 years old (25.6% of men and 37.8% of women). For total corneal astigmatism, WTR also tended to decrease with increasing age: 93.3% of men and 100% of women 20–39 years old versus 20.9% of men and 31.1% of women 80–89 years old. The regression coefficient of both the anterior corneal and total corneal J_0_ vector values analyzed by age were − 0.018 in men and − 0.016 in women (both *p* < .001).

**Conclusions:**

The against-the-rule shift was faster for total corneal astigmatism than for anterior corneal astigmatism and it occurred earlier in men than in women.

**Trial registration:**

Retrospectively registered. Registration number: KC15RISI0241. Registered April 20, 2016.

## Background

Corneal astigmatism can significantly impair visual acuity in phakic and pseudophakic eyes [1, 2]. Uncorrected astigmatism significantly affects distance and near visual acuity and leads to patient dissatisfaction after cataract surgery [3, 4]. As a result, various methods are attempted to reduce corneal astigmatism during cataract surgery in cataract patients and corneal astigmatism.

Toric intraocular lenses (IOLs) have been introduced as a common option for correcting astigmatism without additional corneal refractive procedures, especially in cataract patients with high corneal astigmatism [5]. Visual outcomes with toric IOLs depend on several factors. A precise and predictable IOL power calculation is essential to correct refractive errors properly and to achieve a high level of patient satisfaction after surgery. Previous research has found that the anterior corneal surface shifts from with-the-rule (WTR) astigmatism to against-the-rule (ATR) astigmatism as patients age [6–8]. Recent reports have emphasized the importance of posterior corneal influences on total astigmatism and the need for accurate preoperative measurements of the posterior corneal shape [9]. However, the posterior corneal surface generally has an ATR astigmatism in the optical aspect throughout life. Therefore, it has been proposed that total corneal astigmatism measurements, which include posterior corneal surface contributions, may result in better outcomes than anterior corneal astigmatism measurements when performing toric IOL calculations [9].

Sex-related factors are not generally considered when performing toric IOL axis calculations or in predicting errors in traditional IOL power formulas. However, sex-related differences in ocular anatomy and physiology have been previously investigated [10, 11]. The eyeballs of women are 0.4–0.8 mm shorter than the eyeballs of men [12]. This difference may be why the corneas of women are steeper than those of men [13]. Another study reports that the rate of astigmatism change associated with age in patients diagnosed as having cataracts and is faster in male patients than in female patients [14].

Unfortunately, little is known about how age-related corneal astigmatism changes occur in the anterior and total corneal surfaces in men and women. Thus, the present study analyzed changes in anterior, posterior, and total corneal astigmatism by age and sex in cataract patients. It is our hope that our findings can be used in preoperative toric IOL decisions to improve postoperative outcomes.

## Methods

This retrospective cross-sectional study included 28–89-year-old patients who were diagnosed as having cataracts at Seoul St. Mary’s Hospital (Seoul, South Korea) between May 2009 and July 2013. The inclusion criteria were a healthy ocular anterior segment (as determined by slit-lamp biomicroscopy) and the presence of a visually significant cataract. Patients were excluded if they had systemic disease, previous ocular surgery, irregular astigmatism, glaucoma, traumatic cataract, nystagmus, pterygium, ocular inflammation, dry eye, prior contact lens wear, or poor-quality scans with a Pentacam camera. Patients without corneal astigmatism (< 0.25 D), based on autokeratometer (RK-5; Canon Ltd., Tokyo, Japan) findings, were also excluded. The study protocol was approved by the Institutional Review Board of the Catholic University of Korea (Seoul, South Korea). The study adhered to the tenets of the Declaration of Helsinki and its statement of ethical principles for medical research involving humans.

The patients underwent a complete ophthalmic examination to evaluate cataracts, ophthalmologic disease, and overall ocular health. The anterior segment images, which were obtained by a Pentacam HR rotating Scheimpflug camera (Oculus, Wetzlar, Germany), were used to evaluate corneal astigmatism. The camera is capable of measuring 25,000 data points over the cornea in less than 2 s on the anterior and posterior corneal surfaces and throughout a 180° rotation around the axis of the eye [15]. Pentacam technology provides repeatable and reproducible measurements for the anterior and posterior curvatures [16, 17].

In this study, corneal astigmatism was classified into three categories: anterior astigmatism, posterior astigmatism, and total astigmatism. Anterior and posterior corneal astigmatism were obtained by anterior and posterior keratometric power maps, respectively. The total corneal astigmatism value was obtained by the true net corneal power map. This map uses the real anterior radius, posterior corneal radius, and corneal thickness values and an accordingly modified refractive index. True net power maps were calculated using Gaussian optics formula for thick lenses, which was automatically computed by the Pentacam. First, the refractive values of the anterior surface of the cornea were calculated by using the difference between the refractive index of air (*n* = 1) and the refractive index (*n* = 1.376) for corneal tissue. Second, the refractive values of the posterior corneal surface use the difference between the refractive indices of corneal tissue (n = 1.376) and the refractive index for the aqueous (*n* = 1.336) [18, 19]. All corneal radii were measured in the central 3-mm zone of the cornea [20, 21]. Points on the 3-mm ring are sufficient to analyze astigmatism patterns [21, 22]. Imaging was repeated when images were distorted or improperly taken.

The type of corneal astigmatism was categorized as with-the-rule, against-the-rule, or oblique. When the steepest meridian was between 60° and 120°, then anterior, posterior, or total corneal astigmatism was considered “with-the-rule.” When the steepest meridian was between 0° and 30° or between 150° and 180°, the astigmatism was considered “against-the-rule.” All other astigmatism types were classified as “oblique” [5, 6].

Corneal astigmatism was designated as ATR when the axis of the correcting minus cylinder was within 90 ± 30° and as WTR when the correcting minus cylinder axis was within 180 ± 30° [3, 9]. Oblique astigmatism cases did not show any reliable correlation with age (Table 3) and were not included in the analyses of age-related axial changes.

For vector analysis, we used the following equations [15]:

$$ {\mathrm{J}}_0=-\frac{C}{2}\times \cos\;2\alpha $$

$$ {\mathrm{J}}_{45}=-\frac{C}{2}\times \sin\;2\alpha $$

where C and α represent minus astigmatism power and astigmatism axis, respectively; J_0_ indicates orthogonal astigmatism with perpendiculars of 90° and 180°, with a positive value indicating WTR astigmatism and a negative value indicating ATR astigmatism; J_45_ indicates oblique astigmatism at 45° and 135° [15].

Patients were classified into six age groups, as follows: 20–39 years, 40–49 years, 50–59 years, 60–69 years, 70–79 years, and 80–89 years. These groups were then used to examine any age-related changes in anterior astigmatism, posterior astigmatism, and total corneal astigmatism. In addition, data from men and women patients were separated and individually analyzed.

Data are presented as the mean ± the standard deviation, when applicable. Statistical analyses were performed using SAS statistical software (version 8.0 for Windows; SAS Institute Inc., Cary, NC, USA). The initial analysis showed that J_0_ was not statistically significantly different between the right eyes and left eyes (*p* = 0.130, Wilcoxon signed-rank test); therefore, the right eyes were used for the analyses. The chi square test was used to test differences in WTR and ATR astigmatism distribution for statistical significance between men and women patients and among the six age groups. Data normality was tested with the Kolmogorov–Smirnov test. If normality was rejected (*p* < 0.05), then nonparametric tests were used to determine statistical significance. The Mann–Whitney nonparametric test was accordingly performed to compare the mean values between men and women patients. In addition, Spearman p was used to examine the correlations between the age and J_0_ values. The correlation between age and anterior, posterior, and total corneal astigmatism was determined using power vector J_0_ and linear regression analyses. Statistical significance was defined at a value of *p* < 0.05.

## Results

This study included 415 eyes from 415 patients (217 men and 198 women, with an average age of 63.03 ± 15.91 and 65.52 ± 15.58 years, respectively; age range, 20–89 years). Table 1 summarizes the patient demographics and corneal astigmatism types. The mean anterior astigmatism, posterior astigmatism, and total corneal astigmatism measurements were 0.96 ± 0.60 D (range, 0.30–3.50 D), 0.32 ± 0.17 D (range, − 0.30 D to 0.90 D), and 1.01 ± 0.61 D (range, 0.10–3.20 D), respectively. The parameters of anterior, posterior, and total corneal astigmatism, and age were not statistically significantly different between men and women (*p* > 0.05). Table 2 shows the distribution of eyes according to age and sex.

**Table 1:** Patient demographics

Characteristics	Men	Women	*P* value^a^
Age (y)			
Mean ± SD	63.03 ± 15.91	65.52 ± 15.58	0.117
Eyes (n)	217	198	–
Sex (%)	52.3	47.7	–
Corneal astigmatism (D)			
Anterior			
Mean ± SD	0.96 ± 0.57	0.97 ± 0.63	0.670
Range	0.30, 3.40	0.30, 3.50	–
Posterior			
Mean ± SD	0.32 ± 0.17	0.31 ± 0.17	0.599
Range	0.00, 0.90	− 0.30, 0.80	–
Total			
Mean ± SD	1.03 ± 0.59	0.98 ± 0.63	0.584
Range	0.10, 3.10	0.10, 3.20	–

*D* diopter, *SD* standard deviation

^a^Based on the Student paired *t* test for independent samples between male and female patients

**Table 2:** The distribution of eyes, according to age group and sex

Age Group (y)	Sex (n)	
	Men	Women
≤39	15	13
40–49	32	12
50–59	44	44
60–69	42	42
70–79	41	42
80–89	43	45

Table 3 shows the anterior, posterior, and total corneal astigmatism values transformed into power vector notation and presents them as J_0_ and J_45_ datasets. As age increased, the anterior and total corneal J_0_ vector values became progressively negative (for men: *r* = − 0.541 and *p* < .001, and *r* = − 0.533 and *p* < 0.001, respectively; for women: *r* = − 0.415 and *p* < .001, and *r* = − 0.395 and *p* < 0.001, respectively).

**Table 3:** Corneal astigmatism characteristics converted to vector nomenclature and correlation with age

Parameter	Mean (D) ± SD	Range	*P* value^a^	Correlation coefficient^b^	*P* value
Men					
J_0_ (D)					
ACA	0.078 ± 0.50	− 1.20, 1.70		− 0.541	< 0.001
PCA	− 0.136 ± 0.09	− 0.38, 0.15	0.000	0.316	< 0.001
TCA	−0.057 ± 0.54	− 1.45, 1.55	0.004	− 0.533	< 0.001
J_45_ (D)					
ACA	−0.005 ± 0.24	−0.61, 0.62		−0.083	0.224
PCA	−0.034 ± 0.06	−0.17, 0.20	0.071	0.044	0.516
TCA	−0.039 ± 0.24	−0.74, 0.59	0.107	−0.045	0.363
Women					
J_0_ (D)					
ACA	0.124 ± 0.49	−1.15, 1.75		−0.415	< 0.001
PCA	−0.136 ± 0.09	−0.38, 0.15	0.000	0.266	< 0.001
TCA	−0.002 ± 0.51	−1.54, 1.60	0.009	− 0.395	< 0.001
J_45_ (D)					
ACA	−0.006 ± 0.28	−1.15, 0.90		0.019	0.789
PCA	−0.022 ± 0.06	−0.19, 0.18	0.576	0.065	0.362
TCA	−0.027 ± 0.29	−1.22, 1.15	0.336	0.032	0.657

*ACA* anterior corneal astigmatism, *D* diopter, *PCA* posterior corneal astigmatism, *SD* standard deviation, *TCA* total corneal astigmatism

J_0_ and J_45_ are the astigmatic components

^a^Based on the Mann–Whitney nonparametric test for independent samples between ACA and PCA or ACA and TCA

^b^Based on the Spearman *rho* for the correlation between each parameter and age

**Fig. 1:**
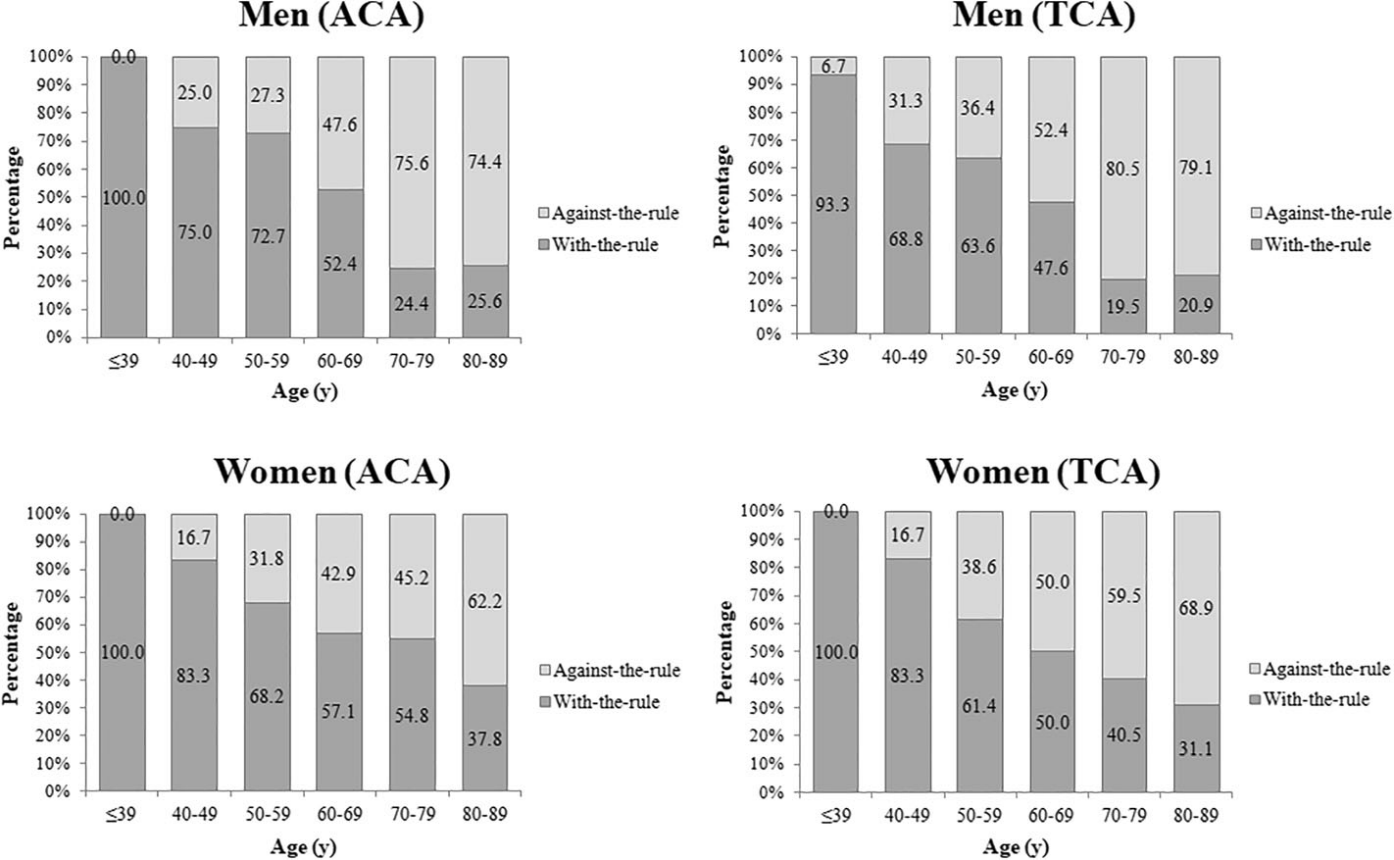
The percentage of with-the-rule type and against-the-rule type in anterior and total corneal astigmatism, according to patient age and sex (J_0_ is the Jackson cross-cylinder, axes at 180 degrees and 90 degrees). ACA, anterior corneal astigmatism; TCA, total corneal astigmatism

Figure 1 shows the percentages of WTR and ATR anterior, posterior, and total corneal astigmatism, based on the patients’ sex and age group. In patients with anterior corneal astigmatism, WTR astigmatism gradually decreased with age in both sexes, beginning at 100% in the 20–39-year age group; and ATR astigmatism gradually and constantly increased in the 80–89-year age group in 74.4% of men and 62.2% of women. For total corneal astigmatism, the percentages of WTR and ATR astigmatism were similar to the changes observed for anterior corneal astigmatism in male and female patients. However, the ATR proportion was higher among patients with total corneal astigmatism than among patients with anterior corneal astigmatism in both sexes. The ATR proportion of total corneal astigmatism was 79.1 and 68.9% in men and women with the 80–89-year age, respectively. The differences between the sexes in the proportion of astigmatism type were statistically significant (chi square test, *p* < 0.001).

**Fig. 2:**
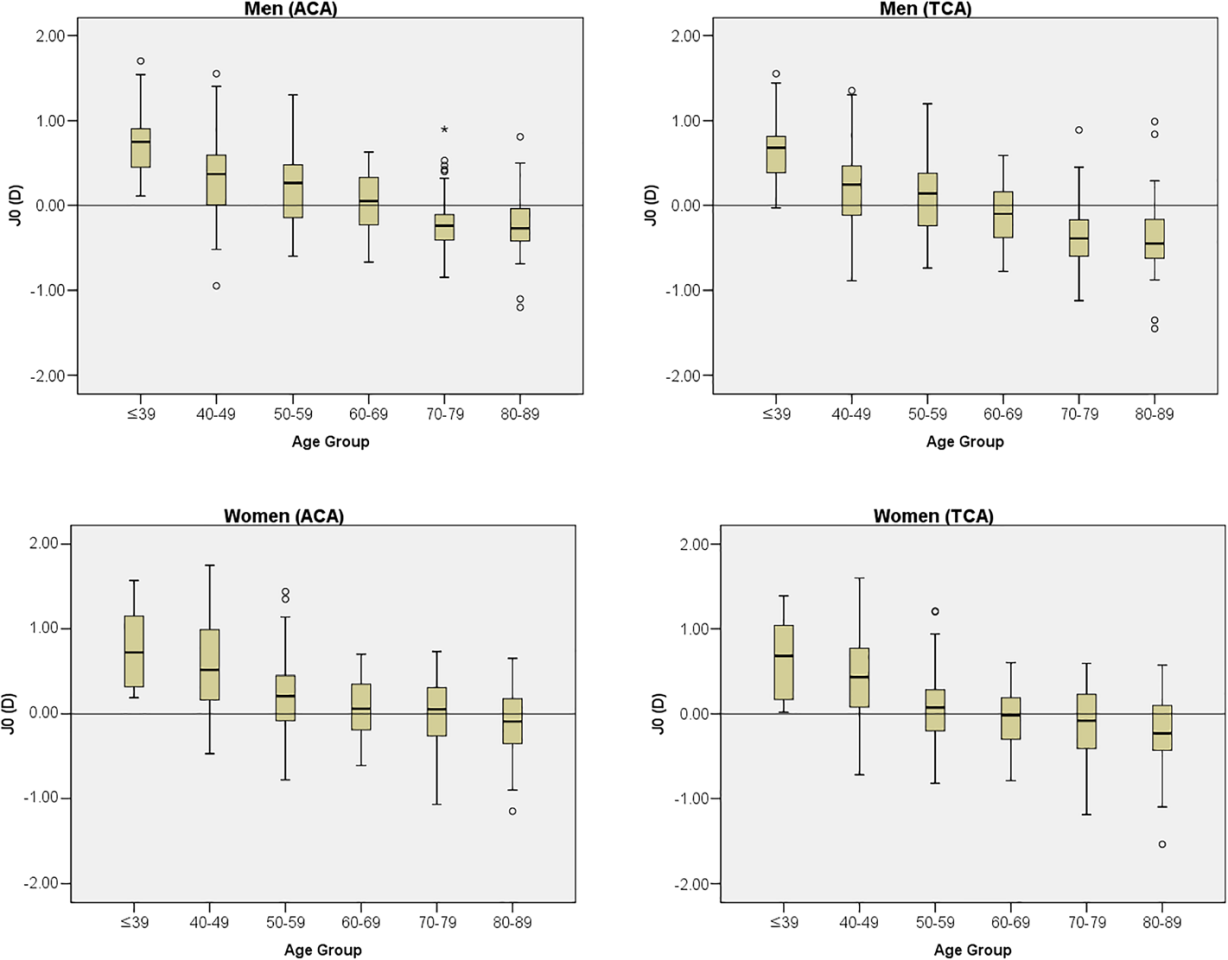
The average J_0_ values, according to patient sex and age (J_0_ is the Jackson cross-cylinder, axes at 180 degrees and 90 degrees). ACA, anterior corneal astigmatism; D, diopter; TCA, total corneal astigmatism

The average J_0_ values according to the patients’ sex and age groups are shown in Fig. 2. The J_0_ value decreased from the 20–39-year age group through the 80–89-year age group in both sexes in the anterior and total corneal surface. The shift from WTR to ATR was faster for total corneal astigmatism than for anterior corneal astigmatism in both sexes. In anterior and total corneal astigmatism, the average J_0_ values decreased faster in men than in women. For anterior corneal astigmatism, the J_0_ became negative more quickly in men (at 70–79 years old) than in women (at 80–89 years old). With increasing age, J_0_ in total corneal astigmatism also shifted toward the negative, which indicated a trend toward ATR at 60–69 years old for men and at 70–79 years old for women.

**Fig. 3:**
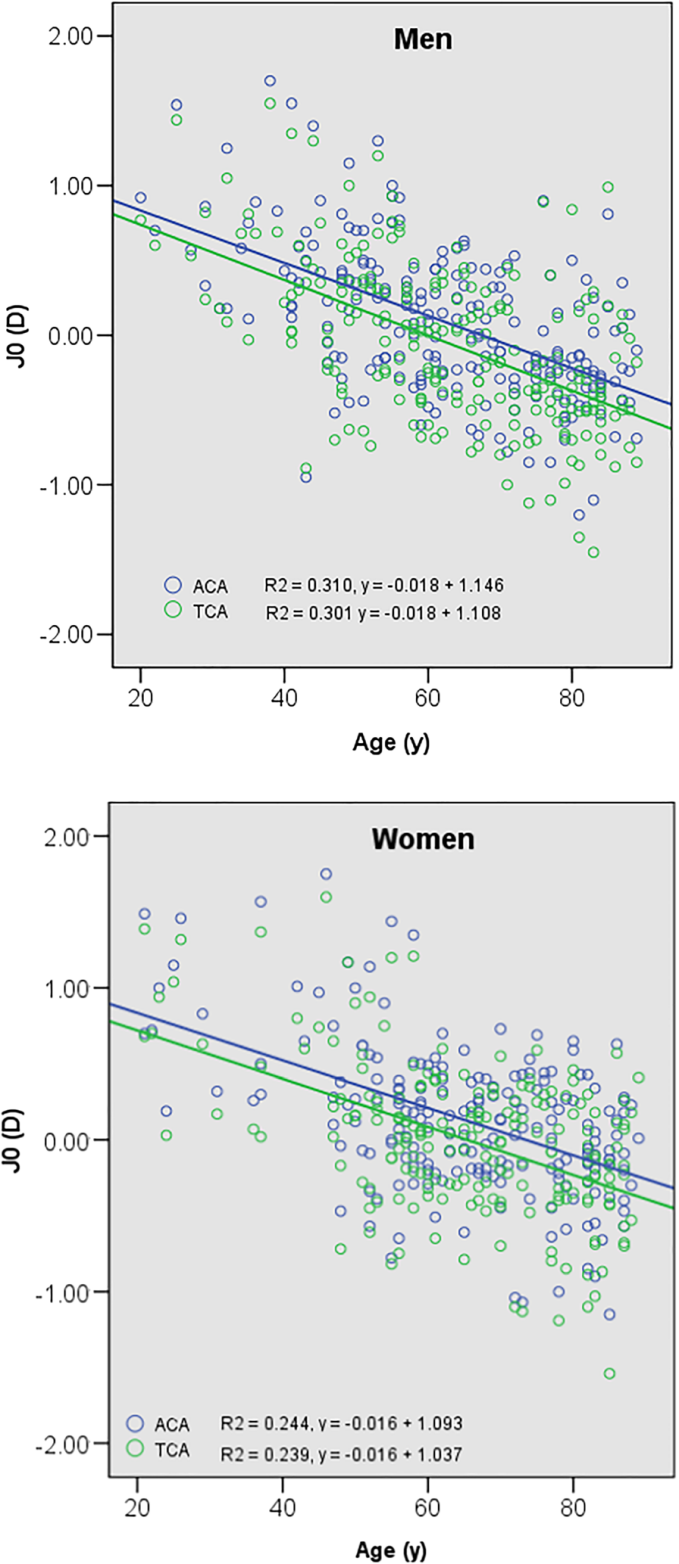
The average J_0_ values for anterior and total corneal astigmatism, according to age in male and female patients. For men, *β* = − 0.018 in both anterior and total corneal astigmatism, and *p* < 0.001 for both types. For women, *β* = − 0.016 in both anterior and total corneal astigmatism, and *p* < 0.001 for both types (*β* is the regression coefficient, based on linear regression analysis; J_0_ is the Jackson cross-cylinder, axes at 180 degrees and 90 degrees). ACA, anterior corneal astigmatism; D, diopter; TCA, total corneal astigmatism

A trend toward WTR to ATR with age was demonstrated by linear regression models; the per-year decrease in age was associated with a decrease in J_0_ of 0.018 in men and 0.016 in women for anterior corneal astigmatism (for both, *p* < 0.001) (Fig. 3). The J_0_ per-year decrease also was 0.018 and 0.016, in men and women, respectively, for total corneal astigmatism (for both, *p* < 0.001) (Fig. 3).

## Discussion

The present study was performed to provide information on sex- and age-related influences in corneal astigmatism using Pentacam scans in cataract patients. We found that the corneal astigmatism in older men tended to shift from WTR corneal astigmatism to ATR corneal astigmatism, compared to older women. In addition, aging influences anterior and total corneal astigmatism differently in older men and women. In our study population, the total corneal astigmatism group had a higher proportion of patients with ATR type, compared to that of the anterior corneal astigmatism group.

Corneal astigmatism, which is reportedly the most common type of astigmatism, changes with advancing age [24–26]. A previous study [21] showed that ATR anterior corneal astigmatism increases with age, but posterior corneal astigmatism remains ATR throughout life in most patients. In the present study, there were differences in the average J_0_ values between anterior and total corneal astigmatism, according to advancing age. We also found that the proportion of patients with anterior corneal astigmatism was different between male and female patients older than 70 years. Among male patients who were 80–89 years old, 25.6 and 20.9% had WTR anterior and total corneal astigmatism, respectively. Among female patients who were 80–89 years old, 37.8 and 31.1% had WTR anterior astigmatism and total corneal astigmatism, respectively. Nemeth et al. [21] reported that ATR astigmatism was present in 7.1% of patients 10–20 years old and in 44.0% of patients older than 81 years. They also reported that most patients had ATR astigmatism in the optical aspect at the posterior corneal surface for all ages examined. However, when analyzing age-related changes in corneal astigmatism, most previous studies did not examine or account for differences between male and female patients or between the anterior corneal surface and total corneal surface [6–8, 21, 22].

Sex-related differences in the anterior corneal surface have been reported in several populations. Differences between men and women in ocular anatomy and physiology have been demonstrated [14]. Previous investigators believed that an ATR astigmatic shift with age depended on fundamental internal factors such as aging of the corneal structure itself [23]. However, the cause of age-related changes in corneal shape remain unclear. Our findings indicated that older men are significantly more likely to have ATR astigmatism in the anterior corneal surface and the total corneal surface than women, which is consistent with previous findings.

Some theories on sex differences in age-related corneal curvature changes have been proposed. Goto el al. [27] reported that simple geometric differences in the globe cannot explain why vertical and horizontal meridians change differently with age in male and female patients. They speculated that corneal curvature changes are caused by axial length changes and intrinsic corneal factors, which are possibly related to the effect of or lack of sex hormones. In support of this theory, differences in corneal stiffness and function between the sexes have been found and attributed, at least in part, to the effects of sex hormones [28, 29]. Interfibrillar spacing of corneal collagen decreases with age while collagen bundles become thicker. Thus, structural changes may alter corneal rigidity and elasticity [27]. Furthermore, it is known that aging decreases sex hormone levels or functional hormone receptors in the cornea [30]. Therefore, sex-related differences in age-related changes in the corneal curvature may reflect how sex hormones influence the cornea.

The present study has several limitations. First, stemming occurred because of its small sample size, particularly for patients younger than 40 years old. Owing to the limited patient number, we were unable to obtain a significant result in this group. Second, data regarding age-related corneal astigmatism did not include factors related to eye size such as the corneal diameter. Third, we did not take into consideration the surgical associated with IOL placement, longitudinal changes, and the effect of cataract severity on corneal structure.

However, to the best of our knowledge, our study is the first study to evaluate statistically age-related changes in patients with anterior astigmatism, posterior astigmatism, and total corneal astigmatism by analyzing rotating Scheimpflug images from male and female patients separately. In cataract patients with astigmatism, an increasing number of surgeons are using toric IOLs to correct astigmatism. Therefore, to obtain adequate refractive and visual outcomes when surgically planning toric IOL implantation, it may be more accurate to consider age-related total corneal astigmatism. In addition, in consideration of the sex-related differences indicated by our results, we suggest that different approaches for men and women patients may be needed when selecting toric IOL and incision. Future research should include longitudinal studies of cataract surgery patients so that individual patterns of corneal astigmatism changes can be better understood and predicted. Further studies on age-related changes in corneal anatomy are also needed.

## Conclusions

In conclusion, our study revealed the proportional differences in ATR astigmatism between patients with anterior corneal astigmatism and total corneal astigmatism, and revealed sex differences in corneal astigmatism with increasing age. The proportion of ATR astigmatism was higher in total corneal astigmatism than in anterior corneal astigmatism, and the ATR type occurred earlier in men than in women. The number of surgeons using toric IOLs to correct astigmatism is increasing; therefore, we performed this study to evaluate the differences in corneal astigmatism with increasing age between the sexes and between the anterior and total corneal surface.

## Abbreviations

ATR: Against-the-rule
IOLs: Intraocular lenses
WTR: With-the-rule
